# Implementation fidelity of a nurse-led falls prevention program in acute hospitals during the 6-PACK trial

**DOI:** 10.1186/s12913-017-2315-z

**Published:** 2017-06-02

**Authors:** Renata T. Morello, Anna L. Barker, Darshini R. Ayton, Fiona Landgren, Jeannette Kamar, Keith D. Hill, Caroline A. Brand, Catherine Sherrington, Rory Wolfe, Sheral Rifat, Johannes Stoelwinder

**Affiliations:** 1 Health Services Research Unit, Department of Epidemiology and Preventive Medicine, Monash University, 553 St Kilda Rd, Melbourne, VIC 3004 Australia; 2 Project Health, 8/150 Chestnut St, Cremorne, VIC 3121 Australia; 3 The Northern Hospital, Northern Health, 185 Cooper Street, Epping, VIC 3076 Australia; 4 School of Physiotherapy and Exercise Science, Curtin University, Kent Street, Perth, WA 6102 Australia; 5 The George Institute for Global Health, Sydney Medical School, The University of Sydney, Camperdown, NSW 2006 Australia

**Keywords:** Falls prevention, Injury prevention, Hospitals, Program evaluation, Implementation fidelity, Quality improvement, Process evaluation, Complex health intervention

## Abstract

**Background:**

When tested in a randomized controlled trial (RCT) of 31,411 patients, the nurse-led 6-PACK falls prevention program did not reduce falls. Poor implementation fidelity (i.e., program not implemented as intended) may explain this result. Despite repeated calls for the examination of implementation fidelity as an essential component of evaluating interventions designed to improve the delivery of care, it has been neglected in prior falls prevention studies. This study examined implementation fidelity of the 6-PACK program during a large multi-site RCT.

**Methods:**

Based on the 6-PACK implementation framework and intervention description, implementation fidelity was examined by quantifying adherence to program components and organizational support. Adherence indicators were: 1) falls-risk tool completion; and for patients classified as high-risk, provision of 2) a ‘Falls alert’ sign; and 3) at least one additional 6-PACK intervention. Organizational support indicators were: 1) provision of resources (executive sponsorship, site clinical leaders and equipment); 2) implementation activities (modification of patient care plans; training; implementation tailoring; audits, reminders and feedback; and provision of data); and 3) program acceptability. Data were collected from daily bedside observation, medical records, resource utilization diaries and nurse surveys.

**Results:**

All seven intervention components were delivered on the 12 intervention wards. Program adherence data were collected from 103,398 observations and medical record audits. The falls-risk tool was completed each day for 75% of patients. Of the 38% of patients classified as high-risk, 79% had a ‘Falls alert’ sign and 63% were provided with at least one additional 6-PACK intervention, as recommended. All hospitals provided the recommended resources and undertook the nine outlined program implementation activities. Most of the nurses surveyed considered program components important for falls prevention.

**Conclusions:**

While implementation fidelity was variable across wards, overall it was found to be acceptable during the RCT. Implementation failure is unlikely to be a key factor for the observed lack of program effectiveness in the 6-PACK trial.

**Trial registration:**

The 6-PACK cluster RCT is registered with the Australian New Zealand Clinical Trials Registry, number ACTRN12611000332921 (29 March 2011).

**Electronic supplementary material:**

The online version of this article (doi:10.1186/s12913-017-2315-z) contains supplementary material, which is available to authorized users.

## Background

In-hospital falls continue to be a major clinical and economic problem for hospitals [1]. They are the most common adverse event in the acute hospital setting [2, 3] and are a source of personal harm [4], increased length of stay (LOS) and increased hospitalisation costs [1]. Consequently, falls prevention is a priority for patient safety activity internationally, with the development of falls prevention best practice guidelines [5–7], the adoption of in-hospital falls as a quality indicator for hospital performance [8–10], and the implementation of a variety of in-hospital falls prevention programs. Despite these efforts, there remains limited evidence to support the effectiveness of such initiatives [11, 12]. Poor implementation fidelity (i.e., the program not implemented as intended) may explain the failure of programs to reduce falls in previous trials.

Falls are a complex problem [13], occurring as a result of complex interactions between physiological, behavioral and environmental factors [14]. This complexity is compounded in the inpatient setting due to the acutely ill nature of many patients, their short LOS and the dynamic nature of the acute hospital ward environment [15]. Therefore, prevention programs are commonly complex, dynamic and patient-orientated, to ensure adequate targeting to the needs of the individual [11]. However, the challenge with testing the effectiveness of such programs is that they are often reliant on the extent to which they have been implemented in practice [16], often referred to in the literature as implementation of intervention fidelity.

Despite repeated calls for the examination of implementation fidelity as an essential component of undertaking trials to evaluate interventions designed to improve the delivery of care [17], it has been infrequently examined in prior falls prevention research [18]. Implementation strategies and levels of implementation fidelity are seldom reported [15, 18]. Only a small number of studies have examined program adherence [19]. The majority of these report less than ideal implementation; with low levels of program adherence [20–22]. Other studies questioned whether limited practice change hindered the program’s effectiveness [23, 24], however they did not report adherence. Therefore the persistent problem of falls in acute hospitals may be due to sub-optimal adoption of falls prevention practices.

Examining implementation fidelity is particularly relevant for complex interventions in multi-center trials, where the same intervention may be implemented and received in different ways across sites [17, 25]. It enables researchers to make valid conclusions about an intervention’s effectiveness or ineffectiveness, ensuring unsuccessful outcomes reflect failure of the intervention and not failure to implement it as intended. A number of frameworks have been developed to examine implementation fidelity [26–30]. The framework proposed by Carroll et al. conceptualizes implementation fidelity as being multifaceted, encompassing both the intervention and its delivery [27]. This framework proposes two key elements: 1) program adherence; and 2) the degree to which adherence was influenced by factors that may have influenced the delivery process [27]. Program adherence, *“the bottom-line measurement of implementation fidelity”* [27] incorporates program content, dose and coverage. Factors that influence program delivery include intervention complexity, implementation strategies, participant responsiveness and quality of delivery.

### Case study: the 6-PACK program

This study concerns the implementation fidelity of the 6-PACK program, a targeted nurse-led multi-component falls prevention program designed specifically for acute hospital wards [29]. It incorporates a nine-item falls-risk tool (the TNH-STRATIFY) [28] and delivery of up to six interventions to patients classified as high-risk based on the tool. However, when tested in a multi-center cluster randomized controlled trial (RCT) (*n* = 31,411 patients), the 6-PACK program did not reduce falls (IRR = 1 · 04, 95% CI, 0 · 78–1 · 37; *P* = 0 · 796) or fall injuries (IRR, 0 · 96; 95% CI, 0 · 72–1 · 27; *P* = 0 · 766) [31]. The primary trial explored reasons for no effect and provided evidence that contamination and confounding were highly unlikely [31]. Given the complexity of falls prevention in the acute ward setting, trial results may have been influenced by the degree to which the program was implemented into practice. The aim of this study was to examine the implementation fidelity— program adherence and organizational support—of the 6-PACK falls prevention program during a cluster RCT [31] to assist with the interpretation of trial results.

## Methods

### Study setting and participants

The methodology and findings of the RCT, conducted between January 2012 and April 2013, are described elsewhere [29, 31]. Intervention wards that participated in the RCT were included in this study—12 acute wards (medical or surgical) from six public hospitals (both metropolitan and regional teaching hospitals) in two states in Australia (Victoria and New South Wales). Participating wards comprised of general medical (*n* = 3), general surgical (*n* = 3), general medical short stay (*n* = 1), specialist medical (*n* = 4) and specialist surgical (*n* = 1). All patients admitted to participating wards were included in this study.

The RCT was a pragmatic trial with the 6-PACK program implemented by ward nursing staff as part of usual care practice. Data from a sample of intervention ward nurses and senior managers from each hospital were used in this evaluation. Sampling procedures are described in the data collection section below.

### The 6-PACK falls prevention program

**Fig. 1:**
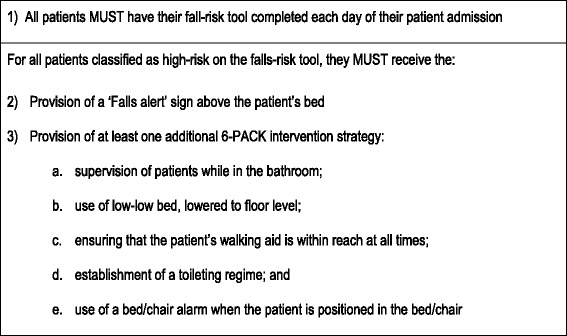
The 6-PACK falls prevention program

The 6-PACK program (Fig. 1) was implemented onto the 12 participating intervention wards during the cluster RCT. Nurses were required to complete the falls-risk tool for each patient on their daily care plan. For patients classified as high falls risk, nurses were to select, document and apply a ‘Falls alert’ sign above the patient’s bed and one or more of the remaining 6-PACK interventions (supervision while in the bathroom; use of a low-low bed, lowered as close as possible to the floor; ensuring their walking aid is within reach; establishment of a toileting regime; and use of a bed/chair alarm when they are in the bed/chair). The selection of 6-PACK interventions were based on the nurse’s clinical judgement of the patient’s needs.

The implementation of the 6-PACK program was managed at a central level (by the research team) and tailored locally by participating hospitals. It was informed by two mixed-methods studies: 1) perceived acceptability of the 6-PACK program (suitability, practicality and benefits) [32] and 2) perceived barriers to, and enablers of, implementation of the 6-PACK program [33]. It incorporated a number of implementation strategies (Table 1), that focused on the training and support of a hospital appointed part-time site clinical leader to facilitate program implementation on intervention wards.

**Table 1:** 6-PACK implementation protocol (strategies for implementation of the 6-PACK program on intervention wards)

		Description
Resources	*Hospital*	Appointment of an executive sponsor (type not specified)
		Provision of 6-PACK falls prevention program equipment^a^: • ‘Falls alert’ signs^b^. One for each inpatient ward bed. • Low-low beds (to be able to be lowered 250 mm from the floor level or lower). A minimum of 1 low-low bed to 3 standard beds on medical wards and 1 low-low bed to 10 standard beds on surgical wards. • Bed/chair alarms. Three on medical wards and one on surgical wards.
		Appointment of a part-time falls prevention site clinical leader^c^ for the 12 month study period. Recommended 0.1 Full Time Equivalent (FTE) for each intervention ward.
	*Ward*	Appointment of two nurses from current permanent staff to act as ward champions to support the site clinical leader and the local assimilation of the 6-PACK program.
Activities	*Hospital*	Integration of the 6-PACK program documentation (risk tool and interventions) into the daily care-plan
	*Site clinical leader*	Attend small interactive face-face group training sessions provided by the research team which included training on the use of the 6-PACK program, leadership, education and change management and provision of a implementation and training guide (two one day sessions, one prior to program implementation and one refresher 6-months post implementation).
		Develop a ‘ReadySetGo’ 6-PACK implementation plan tailored to the hospital and participating wards.
		Deliver small interactive group training sessions to nurses on intervention wards on the use of the 6-PACK program and documentation. Training sessions were based on material provided by the research team but tailored to the needs of the ward by the site clinical leader. A review of site specific case studies were also encouraged. Minimum of two training sessions to be delivered per ward.
		Attend monthly site clinical leader network teleconference meetings with the research team to discuss implementation progress and trouble-shoot implementation challenges (approximately 1 h in length).
		Communicate to ward staff (ward nurses, ward champions and Nurse Unit managers) monthly to provide data on fall event outcomes, risk assessment completion and program adherence.
		Undertake 15-min ward ‘walk rounds’ with ward staff and champions that utilize bedside audit, reminders and feedback: • Weekly for the first month; • Fortnightly for the next 5-months; and • Monthly for the final 6-months.
	*Wards*	Provide monthly data extract reports from the hospital incident reporting database for all participating wards as part of the feedback loop.
		Ward nurses attend 6-PACK program education sessions run by the site clinical leader.
		Ward champions/Nurse Unit managers undertake monthly compliance audits on the use of 6-PACK documentation and nurse’s adherence to the 6-PACK program.

^a^Due to local hospital policies and equipment purchase procedures the make and model of the falls prevention equipment was at the discretion of the hospital. Recommendations were provided to hospitals based on the successful program at The Northern Hospital

^b^ Sign holders for the ‘Falls alert’ signs were recommended but not required

^c^Site clinical leaders were appointed by the hospital, at the discretion of the hospital. Recommendations were provided to hospitals regarding site clinical leader FTE and staff experience, knowledge and skills; however, these were not absolute

Site clinical leaders were provided with a comprehensive 6-PACK implementation, training and monitoring guide. Throughout the trial, support was also provided by the research team (program designer [JK], change management facilitator [FL], project manager [RM] and chief investigator [AB]) on a needs basis via telephone, email and site visits. Need was determined by the site clinical leader or the research team and based primarily on ward program adherence. This included review of complex fall cases, additional training of site clinical leaders on the program and implementation strategies such as audit and feedback and trouble-shooting problems arising with equipment use. Further detail on the 6-PACK program and its implementation can be found elsewhere [29, 31].

### Outcome indicators

An adapted version of the framework developed by Carroll and colleagues was used to assess implementation fidelity. Outcome indicators and data collection sources are summarized in Table 2.

**Table 2:** Outcome indicators and data collection sources for examination of implementation fidelity

Program adherence: the adherence of nursing staff to the use of the 6-PACK program		
Factor	Research question	Data source
*Content*	Did nurses use the individual 6-PACK interventions as designed by the program designers?	Daily structured observation of patient’s bedside Daily audit of patient medical records
*Frequency and duration (dose)*	Did nurses deliver the 6-PACK program as often and for as long as planned, based on targets outlined in Box 1?	Daily structured observation of patient’s bedside Daily audit of patient medical records
*Coverage*	Was the program delivered to all appropriate participants (participant selection)?	As this was a ward based intervention and all patients admitted to participating wards were recruited as part of this study, program coverage was not specifically examined
Organizational support: factors influencing program delivery by nursing staff		
Factor	Research question	Data source
*Hospital and ward resources*	Did hospitals provide the recommended 6-PACK program resources (outlined in Table 1) to allow the intervention wards to carry out the program successfully?	Daily structured observation of patient’s bedside Daily audit of patient medical records Weekly site clinical leader resource utilization diaries Resource and implementation activity compliance log
*Implementation activities*	What implementation strategies (outlined in Table 1) were used by participating hospitals to support, optimize and facilitate the delivery of the 6-PACK program into routine clinical practice?	Daily structured observation of patient’s bedside Daily audit of patient medical records Weekly site clinical leader resource utilization diaries Resource and implementation activity compliance log Attendance at training sessions and network meetings
*Staff perceptions*	How accepting were staff to the implementation of a new falls prevention program? Including staff perceptions on: a) The need for the program b) The alignment of the program with the existing care models and the needs of the patients	Nurse surveys

Nursing practice adherence indicators (program dose) were: 1) falls-risk tool completion each day of admission; and for patients classified as high-risk provision of 2) a ‘Falls alert’ sign; and 3) a sign and at least one additional 6-PACK intervention. As this was a ward-based intervention and all patients admitted to participating wards were recruited as part of this study, program coverage was not specifically examined. Organizational support indicators were: 1) provision of hospital and ward resources (executive sponsorship, site clinical leaders and equipment); 2) implementation activities (modification of patient care plans; training; implementation tailoring; audits, reminders and feedback; and provision of data); and 3) program acceptability among nursing staff.

### Data collection

Data were prospectively collected. Data on program adherence were collected through daily audit of patient care plans and structured bedside observation. Data collection was undertaken by members of the research team (site data collectors) using a standardized tool. Budget constraints meant these data were only able to be collected from the commencement of the cluster RCT until December 2013. Weekly site clinical leader resource utilization diaries detailed time spent on program implementation activities. A member of the research team recorded attendance to site clinical leader training sessions and meetings and kept a log on the compliance of hospitals and wards to resource commitment, care-plan integration and data provision. Nurse perceptions were explored as part of a staff knowledge, attitudes and practices survey, developed by the research team. Nineteen items in the staff survey related to program acceptability scored on a 5-point Likert scale (1 = strongly disagree; 5 = strongly agree). The survey was administered prior to program implementation. The researcher attended handover sessions or designated ward meetings to distribute the survey. Nurses that had worked on an intervention ward for at least 7.5 h per week in the 2 months prior were invited to complete the survey. Completed surveys were placed in sealed boxes collected by the researcher at the end of the 2-week dissemination period. To avoid hierarchical coercion, Nurse Unit Managers were not involved in the distribution of the surveys. The number of surveys distributed to, and returned from, each ward was recorded.

### Data analysis

Descriptive statistics were used to profile the study sample, program adherence and staff perceptions. Adherence to program content was categorized into three levels: adherent, partially adherent and non-adherent. Program dose was defined as a proportion. Data on hospital and ward resources and implementation activities were measured against expectations and/or recommendations and categorized into three level of adherence: adherent, partially adherent and non-adherent. Items were only coded as adherent if the recommendation was completely met. Analyses were undertaken using Stata MP v13 statistical software. Data visualizations of staff survey item responses were created using Tableau Desktop v9.0.

## Results

During the RCT there were 22,670 admissions to the intervention wards representing 17,698 individual participants, as some patients were admitted more than once.

### 6-PACK program adherence

Data on program use were collected from 103,398 daily observations and medical record audits (Table 3). All participating wards were adherent to program content. Overall, 75% of patients had their falls-risk tool completed each day of their admission (range: 52–90%). Of patients classified as high-risk, 79% had a ‘Falls alert’ sign placed above the bed (range: 61–90%) and 63% were provided with a sign and at least one additional 6-PACK intervention (range: 48–74%). Use of 6-PACK program components increased during the RCT period. By the 10th month of data collection 79% of high-risk patients had a ‘Falls alert’ sign plus at least one other intervention (range: 61–95%). Substantial fluctuations in program adherence over the study period and between hospitals were noted (Additional file 1).

**Table 3:** 6-PACK program adherence

	Total (*n* = 103, 398)	Hospital 1: Medical (*n* = 8846)	Hospital 1: Surgical (*n* = 9045)	Hospital 2: Medical (*n* = 8915)	Hospital 2: Surgical (*n* = 8076)	Hospital 3: Medical 1 (*n* = 9231)	Hospital 3: Medical 2 (*n* = 9933)	Hospital 3: Surgical (*n* = 7630)	Hospital 4: Medical (*n* = 10,617)	Hospital 4: Surgical (*n* = 8358)	Hospital 5: Medical (*n* = 9671)	Hospital 6: Medical 1 (*n* = 6657)	Hospital 6: Medical 2 (*n* = 6419)
Program content													
Program content	✓	✓	✓	✓	✓	✓	✓	✓	✓	✓	✓	✓	✓
Program dosage													
Falls-risk score													
Daily completion of falls-risk score, *n* (%)	77,592 (75)	7842 (89)	8161 (90)	7123 (80)	5672 (70)	7625 (83)	7050 (71)	5795 (76)	7897 (74)	4321 (52)	7508 (78)	3748 (56)	4850 (76)
*Patient identified as high risk, n (%)*	*29,330* *(38)*	*3998* *(51)*	*1818* *(22)*	*2651* *(37)*	*2344* *(41)*	*4044* *(53)*	*3221* *(46)*	*669* *(12)*	*2112* *(27)*	*1063* *(25)*	*4309* *(57)*	*1181* *(32)*	*1920* *(40)*
‘Falls alert’ sign													
‘Falls alert’ sign observed to be in place, *n* (%)	23,136 (79)	3425 (86)	1542 (85)	2385 (90)	2049 (87)	3354 (83)	2456 (76)	508 (76)	1626 (77)	737 (69)	2934 (68)	718 (61)	1402 (73)
‘Falls alert’ sign + 1													
‘Falls alert’ sign and at least one additional 6-PACK strategy, *n* (%)	18,445 (63)	2907 (73)	1327 (73)	1972 (74)	1308 (56)	2782 (69)	2096 (65)	407 (61)	1383 (65)	555 (52)	2067 (48)	615 (52)	1026 (53)

✓ Adherent

Program adherence: the adherence of staff to the use of the 6-PACK program. Base on program content and program dosage

Program content: Did nurses use the individual 6-PACK interventions as designed by the program designers?

Program dosage (frequency and duration of program use): Did nurses deliver the 6-PACK program as often and for as long as planned, based on three adherence targets: 1) falls-risk tool completion each day of admission; and for patients classified as high risk provision of 2) a ‘Falls alert’ sign; and 3) a sign and at least one additional 6-PACK intervention strategy

### Organizational support for program delivery

Table 4 summarizes data on the organizational support provided by hospitals during the trial period.

**Table 4:** Organizational support for the implementation of the 6-PACK program: resources and implementation activities

	Hospital 1	Hospital 2	Hospital 3	Hospital 4	Hospital 5	Hospital 6
Hospital and ward resources						
Appointment of an executive sponsor	✓	✓	✓	✓	✓	✓
Provision of 6-PACK equipment as recommended^a^						
‘Falls alert’ sign	✓	✓	✓	✓	✓	✓
Low-low beds	✓	✓	✓	✓	✓	✓
Bed/chair alarms	✓	✓	✓	✓	✓	✓
Site Clinical Leader, FTE per hospital	0.2	0.2	0.1	0.4	0.1	0.1
Appointment of 2 ward champions	✓	✓	✓	✓	✓	✓
Implementation activities						
Hospital activities						
6-PACK program integrated into daily care-plan	✓	✓	✓	✓	✓	✓
Site clinical leader activities						
Attended at SCL training sessions	✓	✓	✓	✓	✓	✓
Developed of ‘ReadySetGo’ plan	✓	✓	✓	✓	✓	✓
Delivered ward staff education and training	✓	✓	✓	✓	✓	✓
Attended monthly SCL network meetings	✓	ϕ	ϕ	✓	ϕ	ϕ
Monthly communication to wards	✓	✓	ϕ	✓	✓	✓
Undertake 15-min ward ‘walk rounds’						
Weekly for the first month	ϕ	ϕ	ϕ	ϕ	ϕ	ϕ
Fortnightly for the next 5-months	ϕ	ϕ	ϕ	ϕ	ϕ	ϕ
Monthly for the final 6-months	✓	ϕ	ϕ	✓	ϕ	ϕ
Ward activities						
Monthly data extract provided	✓	✓	✓	✓	✓	✓
Staff attended education sessions	✓	✓	✓	✓	✓	✓
Monthly compliance audits completed	✓	Φ	ϕ	ϕ	ϕ	ϕ

✓Adherent

ϕ Partially adherent

^a^Recommended allocation of equipment: ‘Falls alert’ signs: 1 per patient hospital bed; Low-low beds: a minimum of 1 low-low to 3 standard beds on medical wards and 1 low-low to 10 standard beds on surgical wards; Bed/chair alarms: three on medical wards and one on surgical wards

*FTE* Full time equivalent

#### Hospital and ward resources

All hospitals provided ‘Falls alert’ signs, bed/chair alarms and low-low beds as recommended. Five of the six hospitals implemented the recommended alarm (Hospital 3 opted for an alternative model). All wards implemented low-low beds that lowered to 250 mm from floor level (as per trial criteria). However, only two of the six hospitals implemented beds that lowered to 100 mm from the floor as recommended.

All hospitals appointed an executive sponsor to support program implementation during the RCT. There was general compliance with the appointment of a site clinical leader. However, three of the six hospitals had less than the recommended 0.1 full time equivalent (FTE) per intervention ward. Hospital 4 provided the greatest site clinical leader resources (0.2 FTE per intervention ward) and Hospital 3 supported the least (0.03 FTE per intervention ward). Hospital 4 experienced a change in their site clinical leader 4 months into program implementation. All hospital wards nominated the recommended number of ward champions, although Hospital 2 experienced a number of staff changes that meant the role was vacant for many months.

#### Implementation activities

All hospitals integrated 6-PACK documentation into the patient care-plan. Each site clinical leader attended two formal face-face training sessions and were provided with the 6-PACK implementation guide. All site clinical leaders were adherent with implementation activities (Table 4), with variability noted in the time allocated to each activity (Additional file 2). Some site clinical leaders spent substantial time undertaking activities not originally envisaged as part of the program implementation, such as administration, email communication and equipment review (range 18–46% of allocated time over the 12-month study period).

#### Staff perceptions

**Fig. 2:**
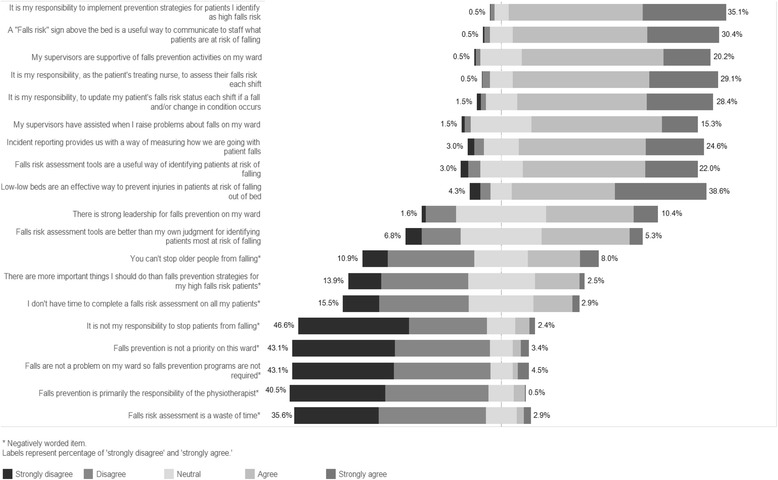
Factors influencing implementation of the 6-PACK program: Staff acceptability of the program

A total of 208 intervention ward nurses (66% response rate) completed the survey. The majority of respondents were qualified registered nurses (85%) and had worked on the participating intervention wards for more than 12 months (68%) with at least five shifts per week (51%). Results have been summarized in Fig. 2. Nurses considered the program components to be important for falls prevention. Falls risk assessment tools were considered useful (74%, aggregate of ‘strongly agree’ and ‘agree’), as were ‘Falls alert’ signs above the bed (87%, aggregate of ‘strongly agree’ and ‘agree’) and low-low beds (83%, aggregate of ‘strongly agree’ and ‘agree’). Survey data revealed that nurses’ believed it was their responsibility to assess the falls risk each shift (87%, aggregate of ‘strongly agree’ and ‘agree’) and implement prevention strategies for patients identified as high falls risk (92%, aggregate of ‘strongly agree’ and ‘agree’).

Nurse responses toward the prevention of falls were less positive. Almost one third agreed that falls cannot be avoided in older people (30%, aggregate of ‘strongly agree’ and ‘agree’). A degree of uncertainty amongst nurses for some items were noted, for example, more than one third neither agreed nor disagreed that there was strong leadership for falls prevention on their ward (38%, ‘neutral’). Moderate variation in staff responsiveness on some survey items between hospitals were observed (Additional file 3).

## Discussion

Overall, an acceptable level of implementation fidelity was observed, though variation was noted between wards and over time. This affirms previously published conclusions, that implementation failure was unlikely to be a key factor for the finding of no-effect in the 6-PACK trial [31].

Program adherence by hospital ward varied across and within hospitals, with adherence levels ranging from 48 to 90%. Substantial fluctuations over time were also noted, with many wards experiencing an increase in adherence as the program continued beyond the initial months. It is likely that these variations are due to a myriad of factors including observed differences in site clinical leaders, their resources and the time allocated to individual implementation activities. The heterogeneity of included wards and patient risk profiles make it difficult to explore these in detail. Nurses have a key role in the implementation of falls prevention programs such as 6-PACK. They care for patients in hospital 24 h a day, 7 days a week, 52 weeks a year. Generally high levels of acceptability of program components was observed among nursing staff, although some concepts demonstrated a proportion of staff with knowledge or attitude limitations that may have limited implementation success.

The completion of the falls-risk tool and provision of falls prevention interventions observed in this study compares favorably with routine practice (e.g. 71% of patients had their risk score updated daily during the RCT, compared with only 13% of patients having their risk score updated at least once during their ward admission in an audit of usual falls prevention practices) [34]. Nearly two-thirds of all high-risk patients received the 6-PACK program as defined in the protocol. While, there may be scope for improvement in program adherence on some participating wards, the characteristics of patients in acute wards (short LOS, complex medical conditions, acute medical instability), mean it may not be realistic to expect 100% adherence. Few previous studies have evaluated adherence to falls prevention interventions in acute hospitals. Therefore, the ability to benchmark the adherence levels observed in this study to prior literature is restricted, and it is difficult to determine what levels of program adherence are actually required to elicit meaningful changes in outcomes.

### Methodological discussion

When interpreting these results, the following methodological limitations should be considered. The provision of 6-PACK interventions was reliant on the daily completion of the falls-risk tool and accurate classification of patients at high-risk. In addition, it does not necessarily reflect optimum practice (the provision of a ‘Falls alert’ sign and all relevant 6-PACK strategies for those identified as high-risk) or allow for the examination of the appropriate use of strategies. The authors acknowledge that qualitative data would provide additional insights into the factors influencing implementation fidelity, yet it was beyond the scope of this study. Considerable but different barriers to implementation appeared to be present in some hospitals/wards during the 6-PACK trial. Further exploration of organizational characteristics, existing infrastructure and surrounding social structures, ward location, the availability, skills and leadership qualities of the local implementation team and other competing priorities experienced by staff should be considered.

## Conclusions

In-hospital falls represent a complex problem that occurs within the complex environment of a hospital ward, often to individuals with complex care needs. Therefore, it is inevitable that examining implementation fidelity is also challenging. Currently there is limited consensus on how best to define and measure implementation fidelity [35]. The framework used in this study provided systematic guidance to measurement, however, it does not provide any direction on how to explore the relationships between its key elements [36].

In this study, while program adherence was not optimal at some participating hospitals, we considered overall implementation fidelity sufficient in the 6-PACK trial. Therefore, implementation failure is unlikely to be a key factor for the observed lack of program effectiveness on fall and fall injuries. This study provides valuable learnings for the development and implementation of future falls prevention programs or other nurse-led safety programs. Given the challenges and costs involved with the implementation of complex health interventions, such as falls prevention programs, examination of implementation fidelity should be an integral part of any evaluation of an intervention’s effectiveness.

## Additional files

Additional file 1: Program adherence of each individual ward by month (DOCX 43 kb)

Additional file 2: Time allocation by site clinical leader to 6-PACK program implementation activities (DOCX 15 kb)

Additional file 3: Influencing factors to implementation of the 6-PACK program: staff acceptability by hospital (PDF 136 kb)

## Abbreviations

FTE: Full time equivalent
LOS: Length of stay
RCT: Randomized controlled trial
