# Supplementary material for: Implementation fidelity of a nurse-led falls prevention program in acute hospitals during the 6-PACK trial
Source: BMC Health Serv Res. 2017 Jun 2;17:383. doi: 10.1186/s12913-017-2315-z (PMC5455084; doi:10.1186/s12913-017-2315-z)
Supplement: Supplementary file 1 — Program adherence of each individual ward by month (DOCX 43 kb) [file 12913_2017_2315_MOESM1_ESM.docx]

**Additional file 1: Program adherence of each individual ward by month**
