# Supplementary material for: Implementation fidelity of a nurse-led falls prevention program in acute hospitals during the 6-PACK trial
Source: BMC Health Serv Res. 2017 Jun 2;17:383. doi: 10.1186/s12913-017-2315-z (PMC5455084; doi:10.1186/s12913-017-2315-z)
Supplement: Supplementary file 2 — Time allocation by site clinical leader to 6-PACK program implementation activities (DOCX 15 kb) [file 12913_2017_2315_MOESM2_ESM.docx]

**Additional file 2: Time allocation by Site Clinical Leader to 6-PACK program implementation activities**

‡6-PACK implementation activities included: Staff training, case reviews, ward ‘walk rounds’, audits reminders and feedback, and ward meetings and communication

†Other activities included: administration, equipment reconciliation, communication with research team
