# Supplementary material for: Implementation fidelity of a nurse-led falls prevention program in acute hospitals during the 6-PACK trial
Source: BMC Health Serv Res. 2017 Jun 2;17:383. doi: 10.1186/s12913-017-2315-z (PMC5455084; doi:10.1186/s12913-017-2315-z)
Supplement: Supplementary file 3 — Influencing factors to implementation of the 6-PACK program: staff acceptability by hospital (PDF 136 kb) [file 12913_2017_2315_MOESM3_ESM.pdf]

Additional file 3: Influencing factors to implementation of the 6-PACK program: Staff acceptability by hospital

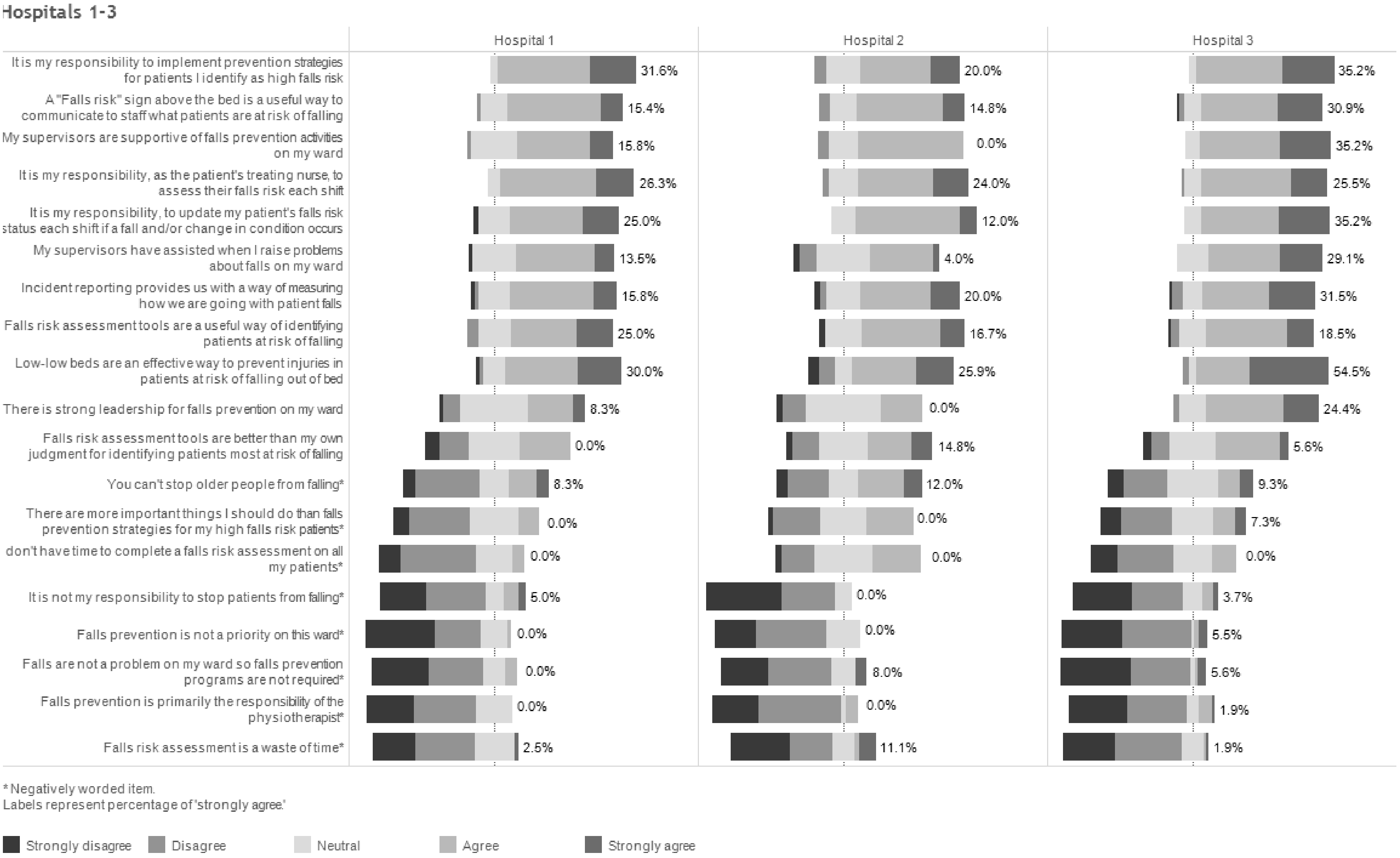

Hospitals 4-6

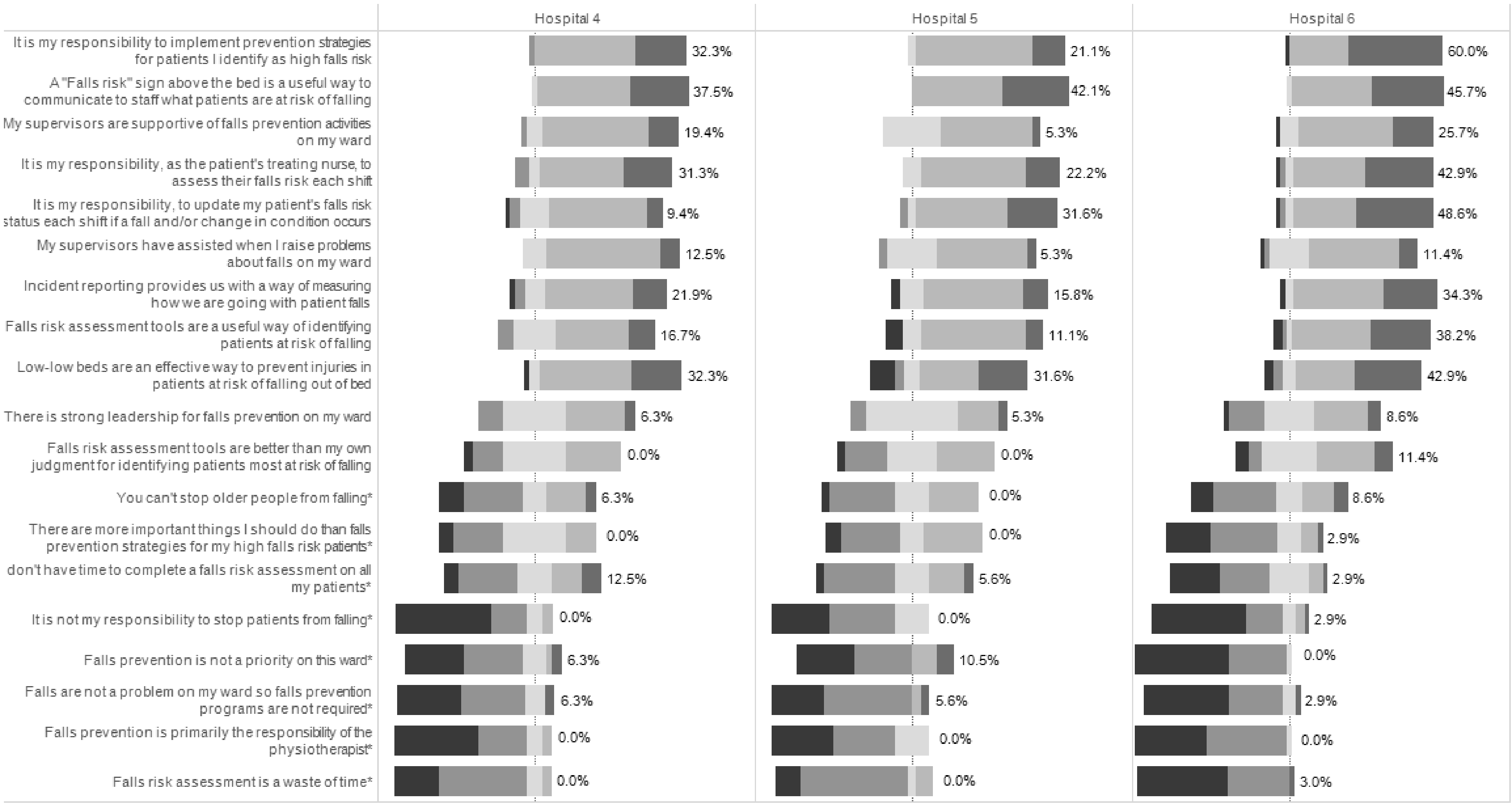

* Negatively worded item.
Labels represent percentage of 'strongly agree.'

Strongly disagree | Disagree | Neutral | Agree | Strongly agree
